# Why did humans surpass all other primates? Are our brains so different? Part 2

**DOI:** 10.1590/1980-5764-DN-2024-0087P2

**Published:** 2024-04-15

**Authors:** Ricardo Nitrini

**Affiliations:** 1 Universidade de São Paulo, Faculdade de Medicina, São Paulo SP, Brazil.

**Keywords:** Primates, Brain, Language, Higher Nervous Activity, Prefrontal Cortex, Culture, Reading, Primatas, Encéfalo, Linguagem, Atividade Nervosa Superior, Córtex Pré-Frontal, Cultura, Leitura

## Abstract

The second part of this review is an attempt to explain why only *Homo sapiens* developed language. It should be remarked that this review is based on the opinion of a clinical neurologist and does not intend to go beyond an overview of this complex topic. The progressive development of language was probably due to the expansion of the prefrontal cortex (PFC) and its networks. PFC is the largest area of the human cerebral cortex and is much more expanded in humans than in other primates. To achieve language, several other functions should have been attained, including abstraction, reasoning, expanded working memory, and executive functions. All these functions are strongly related to PFC and language had a profound retroactive impact on them all. Language and culture produce anatomic and physiological modifications in the brain. Learning to read is presented as an example of how culture modifies the brain.

## RESUMO

A segunda parte desta revisão é uma tentativa de explicar por que apenas o *Homo sapiens* desenvolveu a linguagem. Ressalta-se que esta revisão é baseada na opinião de um neurologista clínico e não pretende ir além de uma visão geral deste tema complexo. O desenvolvimento progressivo da linguagem provavelmente se deveu à expansão do córtex pré-frontal (PFC) e de suas conexões. O PFC é a maior área do córtex cerebral humano e é muito maior em seres humanos do que em outros primatas. Para adquirir a linguagem, diversas outras funções precisavam ter sido alcançadas, incluindo abstração, raciocínio, expansão da memória de trabalho e funções executivas. Todas essas funções estão fortemente relacionadas com o PFC e a linguagem teve um profundo impacto retroativo em todas elas. A linguagem e a cultura produzem modificações anatômicas e fisiológicas no cérebro. Aprender a ler é apresentado como um exemplo de como a cultura modifica o cérebro.

## INTRODUCTION

In the first part of this review, several differences between humans' and other primates' brains were presented. They may explain why other primates do not develop a similar language to ours. Most of the above-cited differences were related to fewer connections between auditory association areas in the temporal lobe and Broca's area, and to the lower development of Broca's area itself in other primates than in humans. Also, consideration was given to the low development of the temporopolar region in non-human primates (NHPs).^1-7^

One may accept these explanations, but the occurrence of primitive oral language in monkeys, such as the vervet and even capuchin monkeys,^8-10^ does not support this contention. Even the possibility that certain sounds emitted by capuchin monkeys are imitations of bird sounds would contradict this explanation. It was also emphasized that other primates can imitate the movements of other animals and humans. This ability would allow them to develop or learn sign language when exposed to it. It has been demonstrated that although chimpanzees were able to learn gestures of the American Sign Language, they could not link words to form a new meaning or use syntax; they could only reach a very low level in this activity.^11,12^ So, there must be other factors beyond those already presented for the almost exclusive development of language in humans. Again, it should be observed that this review is based on the opinion of a clinical neurologist and does not intend to go beyond an overview of this complex topic.

As mentioned earlier, capuchin monkeys can create and use tools.^13^ Our ancestors were able to use tools 2 million years ago.^14^ The ability to draw pictures in caves and mainly to build huts 400,000 years ago demonstrated capacities of planning and abstracting from the real-time to project advantages for the future.^14,15^ For being capable to perform these activities, *Homo* species needed to make use of selective attention, reasoning, abstraction, and executive functions (EFs), which include the programming of the order in a sequence of actions and correction of the steps required for a goal-directed activity.^16^ Working memory (WM) is sometimes included as an executive function or considered a special function. WM is a form of short-term memory, which is essential for the maintenance of a large amount of information while carrying out another task.^15,16^ Tests for WM include the digit span, in which the individual is asked to repeat digits that the examiner speaks randomly at the rate of one number per second.^16^ Usually, we are able to repeat up to 7±2 in the same order and 5±2 in the reverse order. Repetition in reverse order and/or the precise repetition of a phrase are even better tests of other examples of WM.^16^ This ability is crucial for EFs where a sequence of tasks should be performed in a rapid and precise order. It is evident that WM is needed for understanding a paragraph or a long sentence, and to repeat a relatively long phrase. WM may be tested without using language, for example by using the Corsi Block test.^16^ In this tool, nine wooden blocks are randomly pointed by the examiner and the person being examined (or an animal) should reproduce in the same or reverse order.^16^ Although there are controversies, the WM of chimpanzees was about 2±1.^14,15^

As previously explained, much of our knowledge of the relationships between areas of the human brain and normal functions were apprehended by observation of patients who had strokes, tumors, or traumatic injuries to the brain.^17-22^ With this so-called clinical-anatomical or clinical-pathological method, we have learned that the prefrontal cortex (PFC) of the brain is the region where lesions cause the most severe impairments of the above cited activities. Depending on many factors such as the site of the lesion, the subcortical extension of the damage, the age when it occurred, the previous education and personality of the individual, the most common symptoms after PFC lesions are impairments of attention and EFs, disinhibition (or stimulus-bound behavior), reduced empathy and obedience to social and moral rules, and impairment of abstraction and of making appropriate choices in real life. Usually, all these changes in cognition and behavior together do not occur in a single individual. In a few individuals, anything is detected through cognitive tests, but their social abilities or their decision-making is deeply compromised. The absence of symptoms/signs in a few individuals, while other individuals with similar PFC lesions manifest several signs, may mean that PFC underlies functions that are much less "hard-wired".^20^ A few patients, who were previously very correct individuals and fulfilled all their commitments, after a lesion or degeneration of the PFC, begin to behave socially inappropriately and make wrong decisions based almost exclusively on meeting their immediate needs without worrying about the future, as described by Harlow in the famous Phineas Gage case. For his friends and acquaintances, after the accident, he was "no longer Gage".^21^

Cases similar to Phineas Gage have been described in lesions of the ventromedial PFC, which may cause severe disturbances in decision-making and personality.^22^

**Figure 1:**
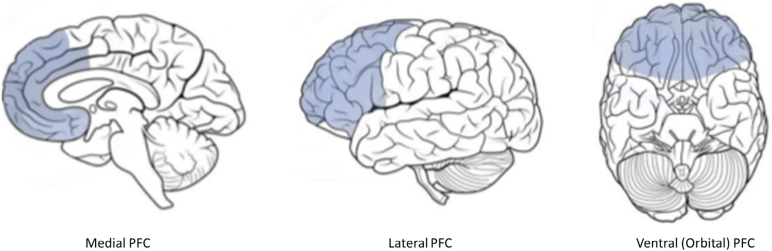
Human prefrontal cortex (PFC) highlighted by blue color^23^.

The PFC is the largest area of the human cerebral cortex (Figure 1)^23^.

It is quite developed in NHPs when compared to other mammals, but it is much more developed in humans. It is not only the higher number of neurons, but mainly the number of synapses due to intrinsic and extrinsic connections.^24-26^ Diffusion tractography revealed a greater proportional volume of frontal white matter networks in humans than in monkeys.^27^

The PFC has reciprocal connections with the unimodal and multimodal associations that are mostly located in the posterior regions of the cerebral cortex, which receive information from the external environment (exteroception), including information from the *where* and from the *what* systems. These connections from the posterior regions of the cerebral cortex reach mainly the lateral PFC through well-developed fascicles.^24,25^ There are also connections with the interoceptive system, which is concerned with collecting information from body sensors and transmitting it to the central nervous system, mainly to parts of the paralimbic and limbic system,^2^ to which the PFC is heavily connected.^28^

Advances in neuroanatomic and neuroimaging methods demonstrated that the PFC is the cortical region with more connections with other cortical areas and with subcortical nuclei such as the basal ganglia and thalamus, through several fascicles.^25^ The PFC is part of many functional systems or networks of connections that explain its participation in activities as broad as planning, control of motor behavior, abstraction, decision-making, adequacy of social behavior, adherence to ethical and moral rules, which are characteristics that define each person's personality.^28,29^

It was also constated that the dorsolateral PFC is the main neural substrate of a network involving several cortical and subcortical brain regions that mediate EFs,^28,30,31^ whereas the ventral and medial PFC are more closely associated with the regulation of emotions, motivation, decision-making, and social behavior.^32^ Also the PFC has reciprocal connections with the systems located in the brain stem and basal prosencephalon that maintain cortical tone.^17,29^

### The evolution of language

It is very complex to determine when language started in primates. Admitting the three stages proposed by Ardila,^33^ the first is formed by noises (grunts), which are a basic communication strategy in chimpanzees (and we still use them in everyday life).^33^ The second is the lexical/semantic stage that we have mentioned is present in vervet monkeys. The last is grammar — the language as a grammatical system. "As linguistic behavior does not fossilize",^11^ it has been very difficult to find out when these stages happened.^33^ Most of the conclusions are based on the technological complexity of tool manufacture, or on the interpretation of the level of abstraction required for creating designs, paintings, and statues.^14,15^ According to these and to the observation of other primates' language, a primitive lexical/semantic system probably appeared long before language as a syntactic system.^11,34,35^ Language as a grammatical system is considered relatively recent and exclusive to *Homo sapiens*.^11,34^ And as all *Homo sapiens* worldwide have this ability, it developed before the great migration from Africa to other continents, which may have occurred some 60–90 thousand years ago.^11,36^ Writing appeared only much more recently (3500 BC).^14^

Language depends on abstraction, which is not an all or none faculty, and grammar may have been developed because of the expansion of WM.^14,15^ Read stated that^5^ in *Homo* species, WM increased progressively from 2±1, which is the chimpanzees' WM, to 3±1, finally reaching 7±2 in *Homo sapiens*. These conclusions were based, as already mentioned, on the complexity of tool manufacture. According to Read, to understand the form "A in the context of B, gives rise to C", it is needed an expanded WM when compared to understanding "A gives rise to B."^15^

It is difficult to credit the development of the grammatical system only to the expansion of WM. Other faculties such as attention, abstraction, and reasoning are also important. Conversely, we have given all credit for this remarkable evolution to the PFC. However, it is necessary to understand that these are not "exclusive functions" of the PFC. All these abilities are distributed in cognitive networks that involve many cortical and even subcortical regions. A good example is what occurs in degenerative lesions involving predominantly the left temporoparietal areas in logopenic primary progressive aphasia.^37^ In this neurodegenerative condition, many patients may be unable to repeat even a simple phrase such as: "the train always whistles when it arrives at the station." The left temporoparietal area is part of the "phonological loop of the WM" that is heavily connected to the PFC, which exerts a top-down control on this loop.^37,38^

Although the development of the PFC and its connections in humans was probably the most important reason for the advent of language, all these abilities such as reasoning, planning, abstraction, EFs, WM, decision-making, and appropriateness of social behavior were profoundly influenced and enhanced by language. Culture and biology allow for the development of humans' higher nervous functions, for which the mediation of language, called semiotic mediation by Luria, is essential.^10,11,22^

Reaching this point, it may be important to think about how language and culture influence the brain.

### Does culture change the brain?

Neuroplasticity is a term commonly referred to in different scenarios, but the underlying concepts are not entirely clear. Would neurogenesis, the formation of new neurons, be one of the significant factors to explain the formidable development of human beings in the first 18 years until reaching adulthood? Probably not. In a study that evaluated more than 100,000 magnetic resonance imaging (MRI) scans obtained from four months of intrauterine life to 100 years of life,^39^ it was possible to observe that the average thickness of our cerebral cortex reaches its apex between 2 and 3 years of age and this increase probably depends much more on the migration of neurons to the cerebral cortex than on neurogenesis.^40^ However, the possibility of neurogenesis can still be raised to explain some phenomena that will be presented later when we discuss learning to read.^41,42^

Studies carried out with animals, initially with mollusks by Eric Kandel's team, revealed that learning and training increase the efficiency of synaptic connections, and also increase the extension of the areas of synaptic connections between two previously connected neurons.^43,44^ The increase in the area of synaptic connections and of synaptic boutons depends on protein synthesis.^44^

Another important mechanism for neuroplasticity is the myelination of white matter fascicles, as described in the development of repetition that occurs with myelination of the arcuate fascicle in the first year of life.^5^ The study with more than 100,000 MRI scans verified that the white matter volume, which largely depends on the number of myelinated fascicles, peaks at around 30 years of age.^37^

Myelination in the central nervous system depends on oligodendrocytes, a glial cell that surrounds axons with a layer of myelin, forming an insulating layer that greatly improves the conduction of nerve stimuli.^45^ Generation and passage of many action potentials along the unmyelinated axonal membrane, when the axonal diameter exceeds a certain magnitude, stimulate conversion of oligodendrocyte precursor cells to mature cells, and the mature oligodendrocytes wrap nearby axons by myelin layers. This link between the passage of more nerve impulses increasing activity of oligodendrocytes and myelination is,^45,46^ in association with increased efficiency of synaptic connections due to training, the most likely explanations for the impact of culture on CNS development. This topic will be further discussed at the end of this article.

### Learning to read and brain development

In investigating the impact of culture on the higher nervous activity development, the study of learning to read is one of the most important. "Learning to read is much more than learning to read".^47^ Here, we will only briefly review the changes that occur in our brain when we learn to read.

To verify whether learning to read increases neurogenesis in the hippocampus,^48^ we investigated the number of neurons in the medial temporal lobe of the hippocampal region of brains of men without cognitive decline from our brain bank.^49^ A comparison was made between brains of individuals with very low formal education (0–4 school years) versus those with higher formal education (≥8 school years). Our idea was based on the knowledge that neurogenesis after birth had been described in the human hippocampus.^50^ However, no difference was found, indicating that the number of neurons in this region probably is not affected by formal education.^48^

Nevertheless, there is at least one study using MRI that showed more gray matter in bilateral angular, dorsal occipital, middle temporal, left supramarginal, and superior temporal gyri in late literates (individuals who learned to read as adults) than in illiterates.^42^ But the authors of that paper did not suggest an explanation for the phenomenon. Higher cortical thickness does not mean higher number of neurons because it may be due to an increase in neurites and synaptic connections (expansion of the neuropile).

Another line of research that showed relevant results was based on neuropsychological, neurolinguistic, and comparative neuroimaging studies between illiterate and literate individuals. This line of research led by the studies of Stanislas Dehaene with the participation of Brazilian researchers, with emphasis on Lucia Braga and her team from the University of Brasilia, managed to demonstrate that the acquisition of literacy transforms the human brain, particularly the systems that are involved in connections between letter vision-phonological analysis.^51-54^

**Figure 2:**
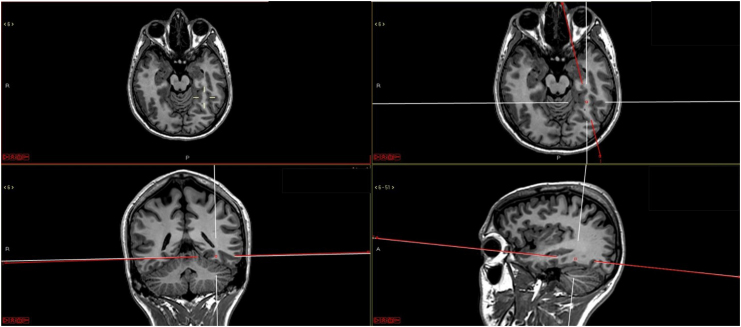
Approximate localization of the Visual Word Form Area in the posterior part of left fusiform gyrus. Courtesy of Leandro T. Lucato.

The posterior portion of the fusiform gyrus (also called the lateral occipito-temporal gyrus) of the left cerebral hemisphere is a region that is naturally important for face and object recognition. When the individual begins to learn to read, this region changes and starts to respond better to letters than to faces (Figure 2), and then it is named the Visual Word Form Area (VWFA).^51-53^

As reading is based on grapheme-phoneme conversion, VWFA communications with the auditory association cortex increase — as demonstrated by tractography studies in which an increase in the posterior temporoparietal portion of the left arcuate fascicle is observed.^51-53^ For the identified phonemes and words that can be spoken, the VWFA connection with Broca's area through the arcuate fascicle is increased.^51^ With learning to read, the interhemispheric connections, mainly through the splenium of the corpus callosum, also increase.^51,53-55^

Changes caused by learning to read on the ventral lexical-semantic route remain to be investigated, but it is probable that modifications have happened.

Functional MRI studies have demonstrated the large web of connections that develop with learning to read.^54^ A positive correlation between literacy levels and hippocampal connectivity with the PFC was also demonstrated.^56^ Even low-educated individuals show better brain white matter microstructure than illiterates.^57^

In addition, with learning to read, the homologous region of VWFA in the right cerebral hemisphere becomes more effective in face recognition.^51.53^

### Language and culture and PFC

Before finishing this topic about the importance of culture on brain development I would like to mention that although the average thickness of our cerebral cortex reaches its apex between 2 and 3 years of age, as already mentioned,^39^ in most countries, young humans reach the status of adulthood at the age of 18 or even 21.

Of course, this old principle is based on the observation that the behavior of an adult human is much more appropriate to live in society than that of a child or even a young teenager. During childhood and mainly throughout adolescence, humans learn several social and moral rules that are based on conventions, which may differ in each society. It is interesting to observe that the PFC is the region of the brain with the richest number of fascicles connecting it with several other cortical and also subcortical areas of the brain.^25^ From childhood to adult life, humans acquire the knowledge of social and moral rules of behavior learned from parents, teachers, books, and other media as well as from the individual's own experience. It is probable that this occurrence at this stage of life, and not before, was related to the development of improved synaptic connections and myelination of many fascicles linking PFC with other regions of the brain. Supporting this latter hypothesis is the knowledge that the latest region of the cerebral cortex to be myelinated is the anterolateral PFC, a region that is especially involved in the evaluation of the hedonic value and emotional implications of behaviors.^58^

Many years ago, we met a 40-year-old male patient who had had a severe lesion of the PFC (mostly involving its ventromedial area) when he was 9 years old.^59^ He survived, got married and had children. His performance in cognitive evaluation was completely normal; he had memorized long excerpts from the Bible and was knowledgeable about local politics. He was considered a cultivated man in his hometown. Nevertheless, he continued to act like a child in most occasions (puerile behavior), with very impulsive or stimulus-bound behavior. From one of his sons, I heard the phrase: "For us, he was like another son of my mother." The patient was unable to learn the social and moral rules that most humans learn during adolescence.^59^ In this period of life, when myelination of the PFC white matter is still ongoing, culture probably has very important impact on the developing fascicles reaching and leaving the PFC.

It does not mean that this is the only way culture influences the developing of PFC and its networks during adolescence and early adult life. A recent study showed that higher cortical thickness of the inferior frontal cortex is positively correlated with the ability of translating feelings or emotions into words.^60^ This ability confers socioemotional advantages and resiliency.^59^ It remains to be elucidated whether the higher cortical thickness is due to learning, but as it may increase with age^60^, culture may be a relevant cause.

The main cognitive and behavioral differences between humans and NHPs may be explained by the development of language and all the advantages it brings. Thinking, abstraction, planning, and executive functions were all greatly improved by language. The achievement of writing with the access to culture provided by it had a profound positive interference with human higher nervous activity. With the improvement in methods of accessing knowledge and information developed in recent decades, we are impressed with the advances in learning that children and everyone who accepted to participate in this digital world are having. And nothing indicates that we are reaching the end of this extraordinary advance in the learning process. This entire new culture will likely change the anatomy and physiology of the human brain, increasing and facilitating synaptic connections, even developing less utilized fascicles and even less probably due to neurogenesis. Unlike natural evolution that takes millennia, the changes we assess will occur in each generation. Although it will probably not be passed on to descendants, it will likely be more easily taught and learned by future generations.

Our higher nervous activities will always change with the external support of culture — hopefully to improve human society.
